# Court-mandated redistricting and disparities in infant mortality and deaths of despair

**DOI:** 10.1186/s12889-025-22221-5

**Published:** 2025-03-19

**Authors:** Alina Schnake-Mahl, Giancarlo Anfuso, Usama Bilal, Neal D. Goldstein, Jonathan Purtle, Stephanie M. Hernandez, Jan M. Eberth

**Affiliations:** 1 Department of Health Management and Policy, Dornsife School of Public Health, Urban Health Collaborative, Drexel University, Philadelphia, PA USA; 2 Department of Epidemiology and Biostatistics, Dornsife School of Public Health, Urban Health Collaborative, Drexel University, Philadelphia, PA USA; 3 Department of Epidemiology and Biostatistics, Dornsife School of Public Health, Drexel University, Philadelphia, PA USA; 4 Department of Public Health Policy and Management, New York University School of Global Public Health, New York, NY USA; 5 Department of Health Management and Policy, Dornsife School of Public Health, Drexel University, Philadelphia, PA USA; 6 Urban Health Collaborative, Drexel Dornsife School of Public Health, 3600 Market St, Room 730, Philadelphia, PA 19104 USA; 7 Department of Microbiology and Immunology, College of Medicine, Drexel University, Philadelphia, PA USA

**Keywords:** Mortality, Congressional districts, Areal units, Health disparities, Redistricting

## Abstract

**Background:**

Health and health disparities vary substantially by geography, including geopolitical boundaries such as United States congressional districts. Every ten years congressional districts for the House of Representatives are redistricted, but occasionally the Courts step in and force states to redistrict gerrymandered congressional maps. Analyses of court mandated redistricting decisions often focus on the distribution of voters by political party and race, but less is known about how health and health disparities are distributed across congressional districts before and after redistricting. In this analysis, we examine how the magnitude of disparities varied *between* and *within* congressional districts in Pennsylvania, before and after the state Supreme Court of Pennsylvania’s decision ordering a redistricting in 2018 that produced less politically gerrymandered districts.

**Methods:**

Using georeferenced vital statistics data from 2013–2015 (before the redistricting), we explore levels of and disparities in infant mortality rates (IMR) and deaths of despair (DoD) using boundaries from before (Congresses 113–115) and after (Congress 116) this redistricting.

**Results:**

Using consistent mortality data (2013–2015) and boundaries from before and after the 2018 redistricting, we find that after redistricting disparities in infant mortality and deaths of despair *between* congressional districts were slightly wider for all educational groups except for those with less than a high school degree, and slightly narrower for all racial-ethnic groups other than for Hispanic and non-Hispanic White populations, compared with before redistricting.

**Conclusions:**

Understanding how disparities vary between and within districts after redistricting can inform our understanding of the relationships between geopolitical boundaries, election processes, and health disparities.

**Supplementary Information:**

The online version contains supplementary material available at 10.1186/s12889-025-22221-5.

## Introduction

The United States has among the highest infant mortality rates of any Organization for Economic Cooperation and Development (OECD) country [1], and has recently experienced stagnating gains, and subsequent reductions, in life expectancy [2]. Within the US, there is substantial variation in these health outcomes: in 2021 infant mortality varied from 2.7 infant deaths per 1,000 live births in North Dakota to 9.4 in Mississippi [3], and life expectancy varied from 71.9 years in Mississippi to 80.7 in Hawaii [4]. Variation in health outcomes, and the magnitude of disparities, are in part determined by the policies and politics that operate in those places. For example, at the state-level, Montez et al. find that more politically conservative economic, social, environmental and health state policy changes are associated with stagnation and declines in US life expectancy [5]. Nations, states, and counties are geopolitical units, or units with both geographic and political meaning [6]. Congressional districts (CDs) are an additional geopolitical unit, but there has been limited use of congressional (or state legislative) districts as a unit of analysis in public health research [7, 8].

Notably, congressional (or state Assembly/Senate) boundaries are not fixed, which creates challenges when performing analysis. Every 10 years, boundaries are redistricted by state legislatures or related commissions following the Decennial Census to account for population change within and between states. Federal requirements for population equality and some limited Voting Rights Act considerations about minority representation exist [9] (which have been weakened by a series of Supreme Court cases over the past two decades [10]), but otherwise states maintain authority to determine congressional boundaries, subject to state and national Supreme Court challenges [11]. States use a variety of different criteria to determine boundaries, including compactness, contiguity, equal population, racial fairness, partisan fairness, preservation of existing geopolitical boundaries or political communities, and/or protecting incumbents [11].

Gerrymandering—or the process of creating boundaries for political advantage, for example by concentrating specific populations, often by race or political party, in a small number of districts to provide an electoral advantage to one group—is a common practice [12]. After *Shelby County v. Holder* [10] determined that the federal approval of voting law changes in states with a history of racial discrimination (known as preclearance) was unconstitutional, a number of states previously subject to preclearance enacted more restrictive voting laws [13]. Together, gerrymandering and the wave of restrictive voting legislation have given disproportionate voting advantage to rural, and generally non-Hispanic White, voters, and weakened the political influence and voting power of urban and racial minoritized populations [14, 15].

Despite these general trends toward more heavily gerrymandered districting since 2010, a small number of states have been required to redraw their maps between Decennial Censuses [16]. For example, in 2018, the Supreme Court of Pennsylvania ordered that the state’s CD boundaries be redrawn when they determined that the 2010 maps adopted by the legislature violated the Commonwealth’s constitution due to extreme partisan gerrymandering [17]. The state legislature and governor could not agree on new boundaries, so the State Supreme Court drew and implemented a new map. Previously, in a process known colloquially as “cracking and packing” [18], the 2010 Pennsylvania boundaries packed Democratic voters into Democratic super majority districts in Philadelphia, Pittsburgh and Scranton, leaving other districts as majority Republican [19]; in the 2016 election Republicans obtained 54% of the statewide vote share but won 72% of congressional seats. In *League of Women Voters of Pennsylvania v. Commonwealth of Pennsylvania,* the plaintiffs contended that state elected officials drew the 2010 boundaries, “with the goal of maximizing the political advantage of Republican voters and minimizing the representational rights of Democratic voters” [20]. The court decision forced a redistribution of voters by district, resulting in a less politically gerrymandered map that produced a shift in elected representatives by party; in the 2018 and 2020 elections, nine democrats and nine republicans won seats [19].

Analysis of redistricting decisions often focuses on the distribution of voters by political party and race, but less is known about how redistricting relates to health and health disparities by district. Populations of similar sociodemographic backgrounds often share health characteristics [21], and there are clear population patterns of health disparities by socioeconomic, racial, and ethnic group [22]. The cumulative impacts of economic inequity and racism (structural, interpersonal, and institutional) are fundamental drivers of health inequities between lower socioeconomic status and racial minoritized populations compared to their higher socioeconomic and non-Hispanic White counterparts [23, 24]. Politics and elected official’s voting patterns are important determinants of health and can bolster or buffer economic inequity and structural racism, yet have not received extensive examination in the public health literature [25–32]. Conversely, the health of populations—and inequities in health outcomes within populations—can influence politics and be determinants of the types of elected officials who are voted into office (e.g., by affecting how people vote and who is healthy enough and alive to vote) [33–35].

In this article, we examine how the magnitude of disparities varied *between* and *within* congressional districts in Pennsylvania before and after the 2018 State Supreme Court decision to redistrict. We seek to understand how redistricting, that attempts to redistribute partisan representation, increases or decreases the concentration of health outcomes. We provide a descriptive analysis to assess how disparities change when boundaries become less gerrymandered, using Pennsylvania, a swing state that had extremely gerrymandered districts after the 2010 redistricting [19]. We include two outcomes, infant mortality (IM) and deaths of despair (DoD: deaths due to drug overdose, alcoholic liver disease, or suicide). These outcomes vary by traditionally studied geopolitical units [29], such as census tracts and county, and by race, ethnicity, and socioeconomic groups [36], are widely available in vital statistics data, and are sensitive to policies decided by state legislatures and Congress [37–41]. This study can help us understand how the distribution of disparities changes after redistricting.

## Methods

### Study design

We examined the consequences of the 2016 Supreme Court of Pennsylvania case that redrew electoral districts after the 2010 maps adopted by the legislature were determined to violate the Commonwealth’s constitution because they were partisan and gerrymandered [17] (See Appendix Fig. 1 for boundary comparisons). The new boundaries applied to the 2018 election and these boundaries were first employed for the 116th Congress, which began in January 2019. Specifically, we compared the IM and DoD mortality rates using the 113th through 115th (years 2013–2018) and the 116th (years 2019–2020, post-re-drawn) congressional boundaries, using deaths that occurred in 2013–2015. In both cases, the number of congressional districts stayed constant at 18. We used the same death data, but different congressional boundaries, to explore how the distribution of deaths in Pennsylvania from 2013–2015 was differently apportioned under two different types of congressional voting district maps. We use the same death data, rather than death data before and after the redistricting, because our intention was to describe how inequity in health was distributed across voting maps rather than to look at the effect of redistricting.

### Data sources and outcomes

We obtained vital registration death records for Pennsylvania from 2013–2015, geocoded to the census tract level [42]. Our primary outcomes were infant mortality and deaths of despair, or deaths due to due to suicide (International Classification of Diseases, Tenth Revision codes X60-84 and Y87.0), chronic liver disease and cirrhosis (codes K70 and K73-4), and alcohol and drug poisonings (codes X40-45, Y10-15, Y45, Y47, and Y49) [43, 44]. Infant deaths included all deaths occurring before one year of age, and for the denominator we used live births from 2013–2015 obtained from vital registration birth records for Pennsylvania and geocoded to census tracts [42]. We limited the DoD analysis to the population aged 25–64, because this population experiences the highest DoD rates [45], and directly age standardized deaths within the 25–64 age range using the 2000 standard population [46]. Denominators for DoD mortality rates were CD level population counts by education level, obtained from 2011–2015 5-year American Community Survey (ACS) pooled estimates. For the 113th-115th congressional boundaries, we pulled congressional district population estimates directly from the ACS, and for 116th boundary estimates we pulled census tract population estimates for 2013–2015 and aggregated them to the 116th boundaries. We calculated IM rates as the number of infant deaths per 1,000 live births, and DoD mortality rates as the number of deaths due to the specified causes per 10,000 persons aged 25–64.

We examined racial-ethnic, and socioeconomic (proxied using educational attainment) disparities in IM and DoD, respectively. Maternal education could not be matched with infant deaths in the vital statistics data so we could not calculate IM disparities by education, and we focused on DoD educational mortality disparities given reports of substantial educational disparities in DoD [47]. For infant mortality, we categorized newborn race and ethnicity into a single ethno-racial construct [48]- (i.e., Hispanic, non-Hispanic White, non-Hispanic Black, non-Hispanic Asian American/Pacific Islander (AAPI), non-Hispanic “Other”) using the race and ethnicity of the mother, or, if unavailable in the vital statistics data, the father [49]. Non-Hispanic “Other” included people who were non-Hispanic Native Hawaiian and Other Pacific Islander alone or multi-racial and were aggregated due to small numbers of individuals and deaths across some congressional districts. We excluded data on the American Indian/Alaska Native population because of small counts (< 0.20% of total state population) and death certificate misclassification [50]. For DoD, we included educational attainment (less than high school, high school, some college/associate, college or more). We removed any observations with missing data for the key variables of interest; for race/ethnicity, data were missing for 1.05% of births and 0.73% of all deaths, and for age and education level, data were missing for 1.08% of all deaths. Of the 2,872 infant deaths and 10,808 DoD included in the vital statistics data for 2013–2015, 321 (11.18%) infant deaths and 407 (3.77%) DoD could not be geocoded and were excluded from the analysis. The deaths that could not be geocoded were socio-demographically similar to the geocoded deaths (see Appendix S2 of Schnake-Mahl et al, 2024 for further details) [7], and any measurement error from undercounting is unlikely to be patterned by redistricting (i.e. missing at random).

### Congressional district boundaries

We used nationally-available crosswalks from the Missouri Census Data Center Geocorr 2018 [51] to aggregate census tract data to the CD level in Pennsylvania. See Schnake-Mahl et al, 2024 for further detail on methods for analysis using congressional districts as a unit of analysis [7]. Briefly, we merged vital statistics mortality data, geocoded to census tracts, with the crosswalks, and aggregated and scaled annual counts of infant deaths, live births, and deaths of despair to CDs to calculate IM and DoD mortality rates by district. We used mortality data from 2013–2015, as this is the most recent mortality data we hade available with local-level geographic identifiers, which allowed us to directly estimate CD-level mortality rates [7].

### Analysis

To explore changes in the distribution of deaths after the redistricting court decision, we mapped the IM and DoD 2013–2015 mortality data to the 2013–2015 boundaries (113th-115th Congresses) and the 2019–2020 boundaries (116th Congress). The 113th-115th Congress boundaries reflect the boundaries later deemed unconstitutional, while the 116th Congress boundaries reflect the new (less gerrymandered) districts. To explore whether there was spatial autocorrelation, and if so, how it changed after redistricting, we computed and compared the global Moran’s I statistic for each set of boundaries [52].

We compared both scenarios using multiple disparity measures that summarize average ordered or unordered group disparities: the mean logarithmic deviation (MLD), mean, standard deviation, and coefficient of variation of mortality rates. The mean, standard deviation, and coefficient of variation are standard measures summarizing the average and variation in mortality rates across all CDs for each racial-ethnic and educational group. The MLD is a relative disparity measure that summarizes the disproportionality between shares of mortality and shares of population, and can be used with ordinal (e.g., education) or categorical (e.g., race and ethnicity) social groups [53]. It is calculated as the weighted average of subgroup-specific mortality rates, using the overall proportion of each subgroup in the population as the weight [53].

We calculated two types of MLDs, calculated separately for each set of congressional boundaries (see Appendix 1 for the MLD calculation equation and example interpretation). First, a *within* CD MLD, in which we calculated an MLD for each CD separately, for IM and DoD, by computing the MLD of IM (using racial-ethnic categories) or DoD (using educational categories) for each social group, and then summed the CD-specific racial-ethnic (or educational) group MLDs to find the MLD for each CD. Finally, we took the median of the CD-specific MLDs to find an overall *within* CD MLD for each set of congressional boundaries. The CD-specific MLDs summarize which CDs have the widest racial-ethnic or educational disparities before and after redistricting, by summarizing the disproportionality between shares of mortality, by racial-ethnic or educational group, and shares of population within each CD. An MLD of zero represents all social groups (race-ethnicity or education) within a CD have the same proportion of deaths as their population proportion, with larger MLDs representing wider disparities between the group rate and overall rate in the CD. The overall MLD summarizes the median within CD MLDs before and after redistricting. To examine the relationship between the MLD disparity and overall IM/DoD rates, we additionally calculated the Pearson and Spearman correlation coefficients for the relationship between each within CD MLDs and the total IM/DoD rates.

Second, we calculated the *between* CD MLD for each racial-ethnic (or educational) group, by calculating the MLD for each racial-ethnic IM (or educational DoD) category separately, using congressional districts as the subgroups. This measure shows which groups have the widest between district disparities in mortality within PA and summarizes the disproportionately between shares of mortality and shares of population across all CDs for each racial-ethnic (or educational) group. An MLD of zero represents, for a specific racial-ethnic (or educational) group, all CDs having the same group-specific mortality rate as the overall population mortality rate, with larger MLDs representing wider disparities. Or put another way, if the MLD is zero for the non-Hispanic Black group, this would mean all CDs would have the same non-Hispanic Black IM rate, and this would be equivalent to the total IM rate for the non-Hispanic Black population in the state.

For all MLD estimates, we calculated the absolute difference between the two congressional boundaries (113th-115th and 116th) estimates to find the change in MLD before and after the redistricting. While it is best practice to calculate both relative and absolute disparities [54], very small MLDs can make the relative measures appear large, so relative measures were excluded to avoid being misinterpreted as indicating very wide differences. All analyses were conducted in R version 4.0.2. Ethics approval was provided by the Drexel University Institutional Review Board, and consent to participate was waived.

## Results

We include Pennsylvania data from 3,200 census tracts, aggregated to 18 congressional districts (CDs). For some districts, the geography and areas covered changed substantially after redistricting (Appendix Fig. 1 and 2). In Appendix Figs. 3 and 4 we show the distribution of the population by race-ethnicity and educational attainment, respectively, for the 113th-115th and 116th congressional boundaries. Two shifts are notable: the non-Hispanic Black population is concentrated in a small number of districts in Philadelphia in Congresses 113–115, but spread among a larger number of districts that cover Philadelphia and the surrounding suburbs in the 116th Congress; and, in and near Pittsburgh, there is a concentration of population with a college degree or more spread across a number of districts in the 113th-115th Congresses, but concentrated in a smaller number of districts in the 116th Congress.

**Fig. 1:**
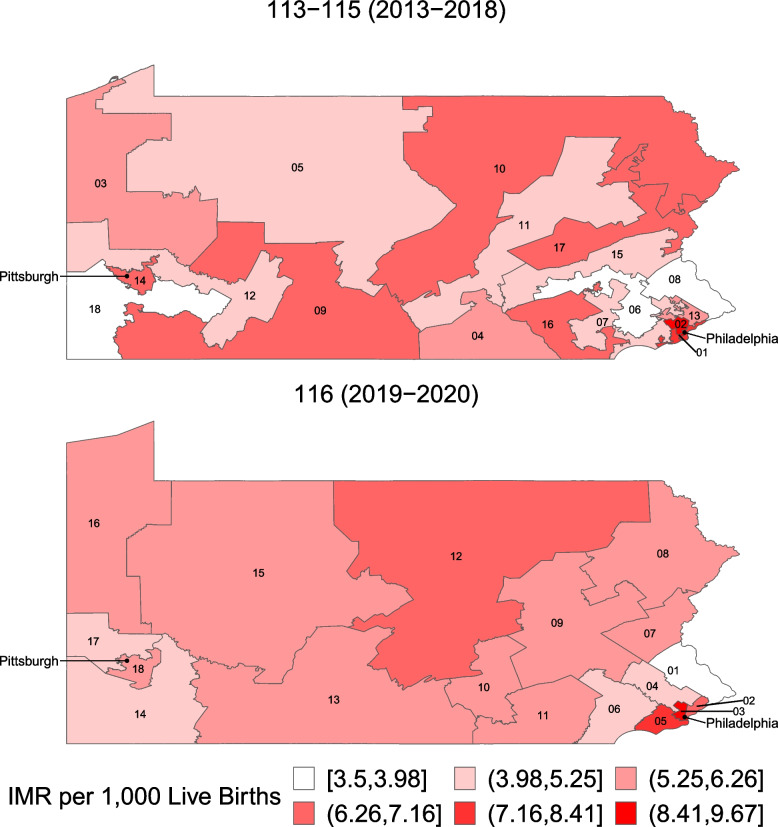
Map of Infant Mortality Rates (IMR) by Congressional District, 113th-115th and 116th Congresses. Footnote: the maps show the infant mortality rate per 1,000 live births, with darker colors representing higher infant mortality rates

**Fig. 2:**
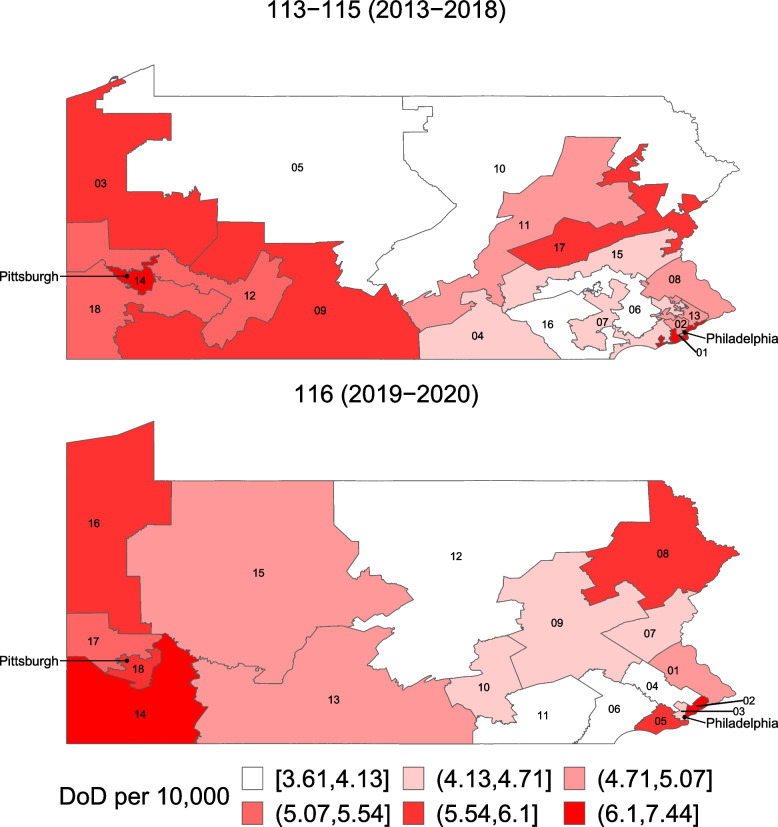
Map of age-standardized Death of Despair (DoD) rates by Congressional District, 113th-115th and 116th Congresses. Footnote: the maps show death of despair rates per 10,000 persons, with darker colors representing higher death of despair rates

We plot the overall IM and DoD rates for each set of congressional boundaries in Figs. 1 and 2, respectively. The mean state IM rates were 5.87 and 5.91 per 1,000 births in the 113th-115th and 116th Congresses, respectively, and the mean state DoD rates were 5.09 and 5.10 per 10,000 persons aged 25–64 in the 113th-115th and 116th Congresses, respectively. The maps show similar rates in Philadelphia and Pittsburgh, with rates tending to decrease in rural areas. There was no evidence of spatial autocorrelation for IM in either Congress (Global Moran’s I = 0.02 and 0.08 for the 113th-115th and 116th Congresses, respectively, *p* = 0.29 and *p* = 0.17, respectively). For DoD, there was evidence of significant autocorrelation in Congresses 113–115 (Global Moran’s I = 0.20, *p* = 0.04), but this decreased after redistricting and no longer showed evidence of spatial autocorrelation (Global Moran’s I = 0.08, *p* = 0.17).

**Fig. 3:**
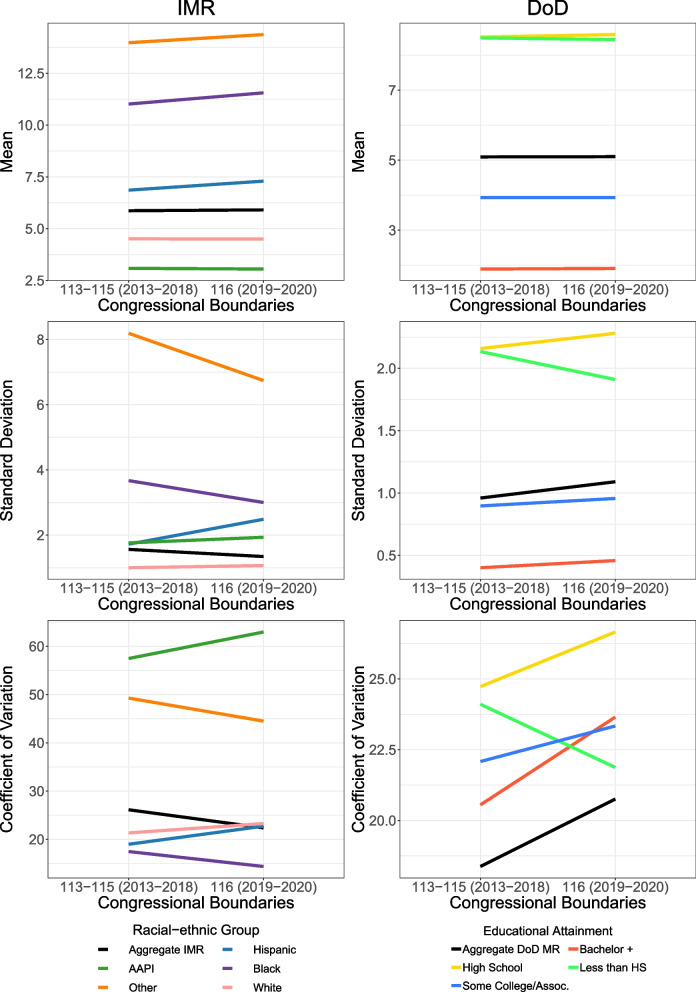
Mean, standard deviation, and coefficient of variation for infant mortality rates and deaths of despair motality rates in Congressional Districts of Pennsylvania in the 113th—115th and 116th Congresses

Figure 3 shows the mean, standard deviation, and coefficient of variation for IM rates by racial-ethnic group and for DoD rates by education, and the mean of these measures across all social groups and CDs. The overall mean IM and DoD rates were the same in both congressional boundaries, but in the 116th boundaries the overall coefficient of variation and standard deviation were smaller for IMR and larger for DoD rates. In the 116th Congress boundaries, average IM rates were higher for Hispanic, non-Hispanic Black, and non-Hispanic “Other” populations, while variability, measured using the standard deviation and coefficient of variation, was smaller for Non-Hispanic Black and non-Hispanic “Other” populations. For all other racial-ethnic groups, there was greater variability in the 113th-115th boundaries than in the 116th boundaries. For DoD by education, mean mortality rates remained mostly the same in the 116th boundaries. Except for the less than high school educated group, there were increases in variability when using the 116th Congress boundaries.

**Fig. 4:**
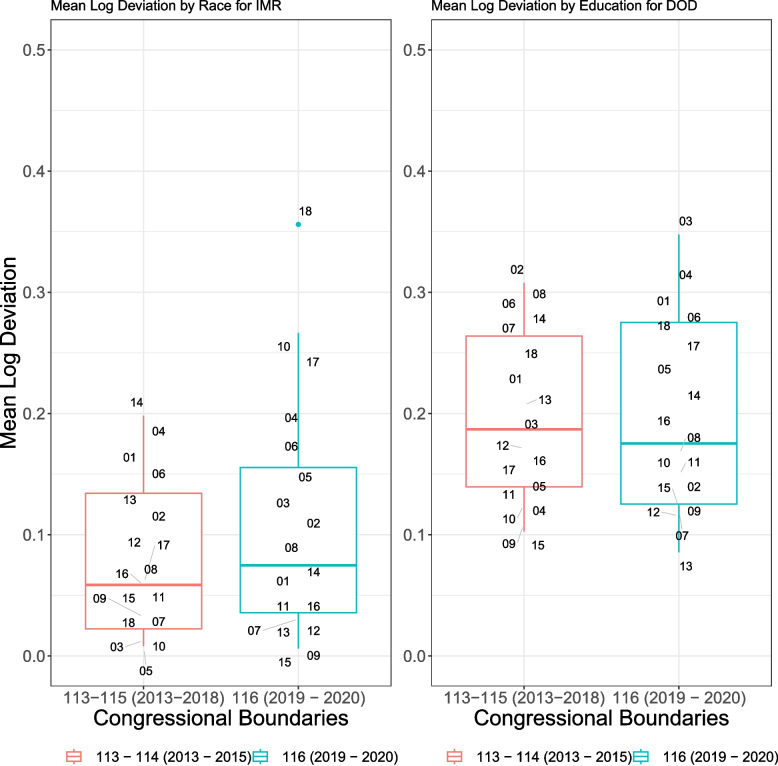
Within Congressional District mean log deviation by race-ethnicity for infant mortality and by Educational attainment for deaths of despair, comparing the 113th-115th versus 116th Congresses. Footnote: The mean logarithmic deviation (MLD) is calculated as the weighted average of subgroup-specific rates, using the overall proportion of each subgroup in the population as the weight, and summarizes the disproportionality between shares of mortality and shares of population

Figure 4 (*within* CD MLDs) shows which CDs had the widest racial-ethnic and educational attainment disparities, for the pre-redistricting (113th-115th Congresses) and post-redistricting (116th Congress) boundaries, for both IM and DoD rates. We avoid comparing the MLDs for each individual CD between Congresses because the geographies covered in each Congress differ substantially (see Appendix Fig. 1 and Appendix Fig. 2). Looking at both educational and racial-ethnic MLDs, we see that disparities differed substantially within CDs across both sets of boundaries. Overall, the median MLD by racial-ethnic group pre-redistricting was 0.059, while the median in the 116th was 0.075, suggesting that overall, there were slightly wider disparities in mortality within CDs after redistricting. For DoD, the overall median within district educational disparities were slightly smaller after redistricting (0.19 to 0.18). For both IMR and DoD, variation in MLDs were larger when comparing the 113th-115th boundaries to the 116th boundaries. The higher median MLD by race for IMR suggests that districts became more racially heterogeneous after redistricting; conversely, the decrease in median MLD by educational group for DoD suggests that districts became less heterogeneous with respect to educational attainment. The Pearson and Spearman correlation coefficients between the within CD MLDs and total IM/DoD rates showed weak correlations (0.06 and 0.09, respectively, for IMR, and 0.12 and 0.07, respectively, for DoD MR), suggesting no clear relationship between the size of disparity and overall mortality rates.

Table 1 (the *between* CD MLDs) shows which racial-ethnic and educational attainment groups have the widest between district disparities in mortality for each set of congressional boundaries. We found that the between CD racial-ethnic MLDs were close to zero for most racial-ethnic groups other than Non-Hispanic Asian (suggesting equal spread across districts) pre-redistricting, and minimally different between the two CD maps (largest absolute difference was −0.019 for non-Hispanic “Other”); disparities for non-Hispanic Black infants were slightly narrower, while disparities were slightly wider for Non-Hispanic White and Hispanic infants in the post-redistricting map compared to the pre-redistricting map. In both Congresses, between CD disparities were widest for non-Hispanic AAPI and non-Hispanic “Other”, and lowest for non-Hispanic Black, and Hispanic populations. For educational between CD disparities in DoD, we found disparities were slightly wider post-redistricting for all groups except for less than high school (largest absolute difference was 0.008 for high school and college educated populations). For both sets of congressional boundaries, disparities were the widest for the High School group, followed by the less than high school group for the 113th–115th boundaries and the bachelor’s degree or more group for the 116th boundaries. However, all MLDs were close to zero, suggesting relatively equal spread between districts.

**Table 1:** Between Congressional District Disparities (Mean Logarithmic Deviation) between 113th-115th and 116th Congresses, for infant mortality by race-ethnicity and deaths of despair by educational attainment

	Congress 113—115	Congress 116	Absolute Difference
**Racial-Ethnic category specific IM disparities**			
Hispanic	0.018	0.025	0.007
Non-Hispanic White	0.022	0.028	0.007
Non-Hispanic Black	0.018	0.012	−0.006
Non-Hispanic Asian/ American Pacific Islander	0.211	0.210	−0.001
Non-Hispanic “Other”	0.105	0.086	−0.019
**Education Attainment specific DoD disparities**			
Less than High School	0.028	0.024	−0.004
High School	0.030	0.037	0.008
Some College/Associate's Degree	0.024	0.025	0.001
Bachelor/Master/Doctoral Degree	0.021	0.029	0.008

The mean logarithmic deviation (MLD) is calculated as the weighted average of subgroup-specific rates, using the overall proportion of each subgroup in the population as the weight, and summarizes the disproportionality between shares of mortality and shares of population. Absolute difference is calculated as the MLD in the 116 minus the 113–115 MLD

## Discussion

We describe overall rates and racial-ethnic disparities in infant mortality rates and socioeconomic disparities in rates of deaths of despair across Pennsylvania congressional districts, comparing rates using the 113th-115th congressional district boundaries to rates using the post-redistricting 116th district boundaries, while holding the data constant. The redistricting reduced partisan gerrymandering, and we observed the largest differences in the distribution of rates in the areas close to Philadelphia and Pittsburgh.

Concentrating populations with similar health outcomes results in areas with, for example, worse overall health than in other CDs but limited disparities, and this could result in a form of health gerrymandering. This may help explain why there was a widening of within CD health disparities by race-ethnicity between the politically gerrymandered districts in the 113th-115th Congresses and the post-redistricting 116th Congress. Districts were more homogeneous on health and sociodemographic characteristics in the gerrymandered districts and then became more heterogeneous after redistricting. Specifically, disparities between districts were slightly narrower for the non-Hispanic Black population after redistricting (as the Black population was distributed across districts more fairly), indicating that infant deaths were more equally split between CDs, whereas a large majority of the Black population, and Black infant deaths, were concentrated in a small number of districts prior to redistricting. These findings support the “cracking and packing” gerrymandering concept [18], in which the non-Hispanic Black population was packed or concentrated in a small number of districts, and then after redistricting was spread across additional districts. Alternatively, while overall educational DoD were quite equally spread across districts, after redistricting within district disparities were slightly narrower, while between district disparities were very slightly wider, for all education groups other than the less than high school educated group. This suggests deaths of despair rates by education level became more homogenous within CDs and slightly less equally distributed between CDs after redistricting, for most educational groups, while becoming slightly more equally distributed between CDs for the less than high school educated group. As voting behavior has become increasingly partisan by education level [55], and deaths of despair are increasingly concentrated among the population without a high school degree [2], creating more partisan districts may require greater educational heterogeneity generally, while resulting in a more equal dispersal of the less than high school educated population. Together, our findings suggest that redistricting for political equity (e.g., less gerrymandering) may have complicated implications for the distribution of health, as greater political equity may produce more disparate health outcomes within districts for some sociodemographic groups and outcomes (e.g., education and DoD) while more equally distributing others (e.g., race and IMR) [56].

While our findings are primarily pertinent to understanding the overall distribution of health disparities based on the level of partisanship within the boundaries of congressional districts, the findings may also impact individual congresspersons’ and collective congressional activity. While wider disparities within a district may make representatives more attuned to supporting policies to shrink disparities [57], lack of disparities among a representative’s constituents may have the opposite impact, making representatives less concerned with the impacts of congressional policy on disparities. Alternatively, narrow disparities but poor overall rates in a district may encourage representatives to support universal policies without targeting sub-populations. This is because policy makers often want [58] and are attuned to locally relevant evidence and data [57, 59, 60], and policy makers are more likely to be concerned when a public health challenge is more severe, according to epidemiologic data, in their geopolitical jurisdiction [57, 61]. Further, disparities spread out across numerous districts may encourage congresspersons to work together to support policies to address health inequities.

An increasing body of research in the area termed “political epidemiology” explores the impacts and associations between political factors and health outcomes and disparities, building on political science policy feedback work [62, 63]. For example, Rodriguez finds that excess mortality among US Black populations dampens their political voice, specifically that Black excess mortality reduced the 2004 voting age population by 1.7 million [33]. In turn, the reduction in the voting power of Black populations may contribute to further excess mortality for Black and other minoritized populations, because voting, and who is elected to office, shape voting on policies that impact the structural, social, and economic conditions that give rise to inequitable health outcomes, including excess mortality [33, 64]. Counterintuitively, districts becoming less partisan and more sociodemographically diverse may reduce the political power of a single racial-ethnic group, such as the Black population, diminishing the group’s political power and the elected representative’s focus on the specific health needs of that population [29, 65, 66]. Related, lower state level voting representation of poorer voters is associated with less generous state welfare spending [67], and less generous welfare spending is disproportionally harmful for the lowest income populations [68]. Together, this leads Rodriguez to argue that “excess mortality in marginalized populations could be both a cause and an effect of political processes “ [33]. Alternatively, political processes and court decisions can also improve health outcomes for marginalized populations; Rushovich et al. found improvements in Black infant mortality after the Voting Rights Act, with larger reductions in Black infant deaths in counties required to remove voter suppression policies than in counties without such requirements [28]. Though our article uses descriptive analysis and does not specifically assess the effects of political processes, court cases forcing redistricting of gerrymandered districts may be one approach to redressing power inequities and consequent health effects. Future research can examine the causal effects of processes and criteria to draw CD boundaries on variation in health disparities within and between district boundaries, can consider impacts on additional mortality types and other health metrics, and incorporate analysis of both short and longer-term health effects [69]. Additional questions about feedback mechanisms between policies, voting patterns, and the production of health disparities are well suited for systems science methods, and deserve further investigation. Our article builds on this political epidemiology research, as well as research that considers political processes and structures in epidemiologic research [69], to understand how differences in the configuration of congressional districts result in differences in the distribution of two mortality outcomes, infant mortality and deaths of despair.

We note several limitations. Namely, data challenges including: a small amount of mortality data missing geographic identifiers; potential death certificate misclassification for causes of death, and educational categories; and potential misclassification of deaths to census tracts during the geocoding process, as well as grouping of ethno-racial groups due to small sample sizes. Our analysis used mortality data from 2013–2015, but results may differ using other years of data, given that in the U.S. deaths of despair rose between 2010 and 2017 [45], while during the same period infant mortality rates slightly decreased [70]. Finally, the analysis was limited to a single swing state, and findings may not be generalizable to states with more partisan voting constituencies.

## Conclusions

This article provides a descriptive analysis of infant mortality and death of despair rates and disparities before and after the Pennsylvania Supreme court redistricting decision in 2018. After the redistricting, between district disparities were narrower for the non-Hispanic Black, non-Hispanic AAPI, and non-Hispanic “Other” populations but wider for other groups, including all education groups other than less than high school educated. These findings suggest that as congressional districts become less politically gerrymandered, the distribution of health disparities across congressional districts may widen for some groups, while narrowing for others. These findings have implications for our understanding of political election processes and health disparities and provoke new causal questions about the effects of redistricting on population health and health disparities.

## Supplementary Information

Supplementary Material 1.

## Data Availability

No datasets were generated or analysed during the current study.
