# Supplementary material for: Court-mandated redistricting and disparities in infant mortality and deaths of despair
Source: BMC Public Health. 2025 Mar 19;25:1058. doi: 10.1186/s12889-025-22221-5 (PMC11921522; doi:10.1186/s12889-025-22221-5)
Supplement: Supplementary file 1 — Supplementary Material 1. [file 12889_2025_22221_MOESM1_ESM.docx]

**Appendix**

**Appendix 1: Example MLD calculation**

| **MLD Equation:**  $MLD=\sum p_{j}(-ln r_{j})$  **Within CD MLD:**  We calculated within CD MLD’s separately for each Congress (113^th^/115th and 116^th^). Using IM and racial-ethnic groups as an example, we calculated an MLD for each CD, where $p_{j}$ is the proportion of births in each racial-ethnic group, and where $r_{j}$= $\frac{y_{j}}{\mu}$ where $y_{j}$is the IM rate in racial-ethnic group *j,* and $\mu$ is the total CD population IM rate.  We then took the weighted sum of the within CD racial-ethnic group MLDs to find the MLD for each CD. Finally, we calculate the median of the CD specific MLDs to find an overall MLD for each Congress.  Example interpretation:  An MLD of 0 in CD 1 would mean that in CD 1, if the Black births were 10% of the CD births, then 10% of all deaths in the CD would be among Black infants, and if non-Hispanic White births were 20% of all CD births, then 20% of all deaths in the CD would be among non-Hispanic White infants, etc. for all other racial-ethnic groups in the CD.  **Between District MLD:**  Using the IM for non-Hispanic Black population as an example, we calculated an MLD for each racial-ethnic group separately, where $p_{j}$ is the proportion of Black births in CD *j*, and $r_{j}$ is the ratio of the Black IM rate in CD *j* relative to the total Black IM rate in Pennsylvania. We then took the sum of the racial-ethnic group MLDs across CDs, to find each racial-ethnic group specific MLD for all of Pennsylvania.  Example Interpretation:  A MLD of 0 for the non-Hispanic White population means that non-Hispanic White infant deaths are equally spread across all CD’s, so that the proportion of deaths among White infants overall is equal to the proportion of births among White infants in each CD. |
| --- |

**Appendix Figure 1: Comparison of Boundaries for the 113^th^-115^th^ (2013-2015) Congresses and the 116^th^ Congress (2019-2020)**

**Appendix Figure 2:** **Share of census tracts changing between CDs from the 113^th^-115^th^ (2013-2018) Congresses to the 116^th^ Congress (2019-2020)**
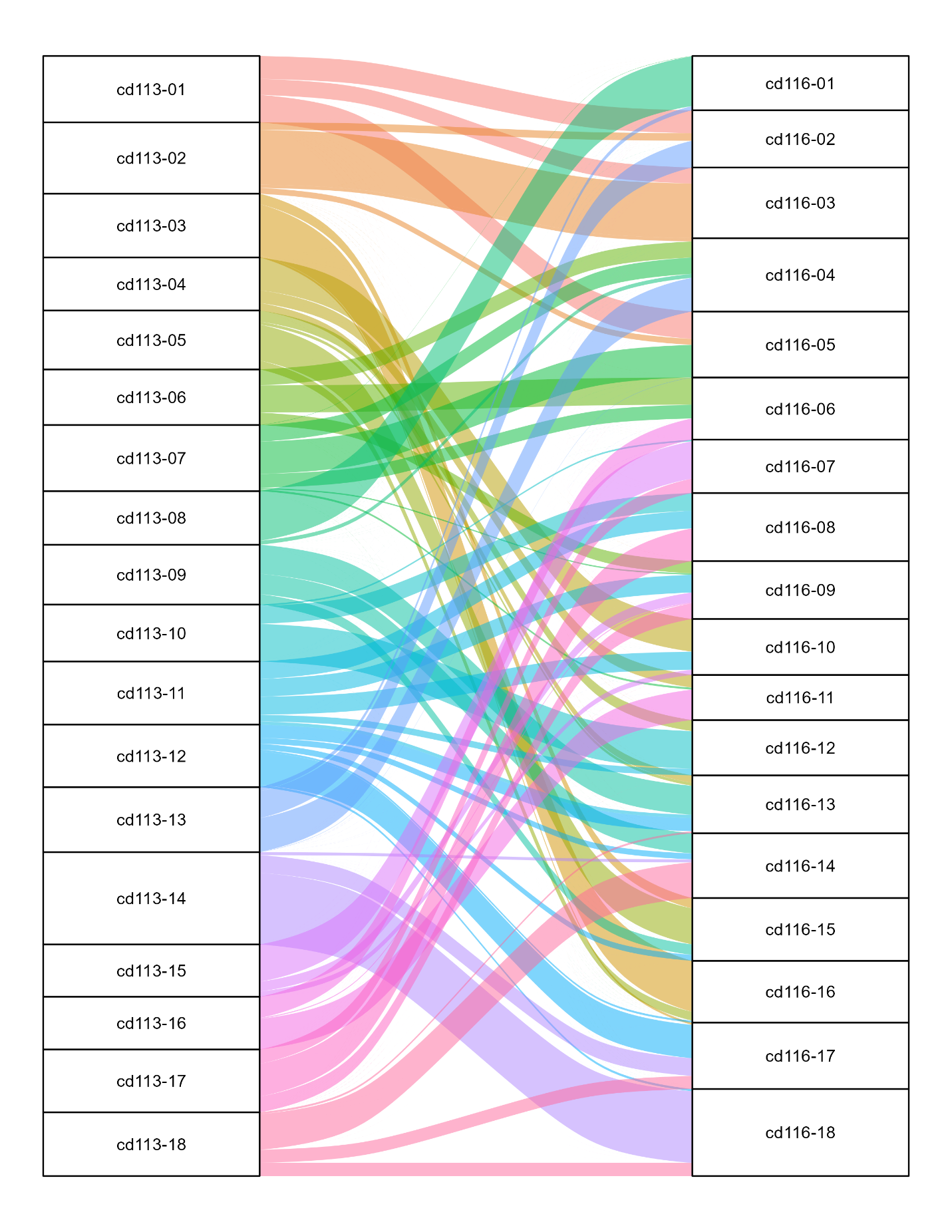


**Appendix Figure 3: Racial-ethnic Distribution of Live Births in Pennsylvania for the 113^th^-115^th^ and 116^th^ Congressional Boundaries**

**Appendix Figure 4: Educational Attainment Distribution of Pennsylvania Residents for the 113^th^-115^th^ and 116^th^ Congressional Boundaries**
